# Rapid Encoding of New Memories by Individual Neurons in the Human Brain

**DOI:** 10.1016/j.neuron.2015.06.016

**Published:** 2015-07-01

**Authors:** Matias J. Ison, Rodrigo Quian Quiroga, Itzhak Fried

**Affiliations:** 1 Centre for Systems Neuroscience, University of Leicester, Leicester, LE1 7QR, UK; 2 Department of Engineering, University of Leicester, Leicester, LE1 7RH, UK; 3 Department of Neurosurgery, David Geffen School of Medicine and Semel Institute for Neuroscience and Human Behavior, University of California Los Angeles, Los Angeles, CA 90095-7039, USA; 4 Functional Neurosurgery Unit, Tel-Aviv Medical Center and Sackler Faculty of Medicine, Tel-Aviv University, Tel-Aviv, 69978, Israel

## Abstract

The creation of memories about real-life episodes requires rapid neuronal changes that may appear after a single occurrence of an event. How is such demand met by neurons in the medial temporal lobe (MTL), which plays a fundamental role in episodic memory formation? We recorded the activity of MTL neurons in neurosurgical patients while they learned new associations. Pairs of unrelated pictures, one of a person and another of a place, were used to construct a meaningful association modeling the episodic memory of meeting a person in a particular place. We found that a large proportion of responsive MTL neurons expanded their selectivity to encode these specific associations within a few trials: cells initially responsive to one picture started firing to the associated one but not to others. Our results provide a plausible neural substrate for the inception of associations, which are crucial for the formation of episodic memories.

## Highlights

• Contextual associations were used to model the formation of new memories
• Human single neurons changed their firing patterns to encode new associations
• Changes occurred at the exact moment of learning, even after single presentations
• The rapid speed of neural changes is compatible with episodic memory formation

## Teaser

Ison et al. recorded from single neurons in the human brain while patients learned contextual associations. They found that neurons change their firing to incept new associations even after one single presentation, thus providing a plausible mechanism underlying memory formation.

## Introduction

Neuroimaging investigations in humans and behavioral studies of neurological patients have substantiated the importance of the medial temporal lobe (MTL) for episodic memories (Davachi, 2006; Eichenbaum, 2004; Eichenbaum et al., 2007; Moscovitch, 1994; Squire et al., 2004; Tulving, 2002). Furthermore, neurophysiological and lesion studies in animals have shown that the MTL is involved in the encoding of associations (Bunsey and Eichenbaum, 1996; Day et al., 2003; Kahana et al., 2008; Sakai and Miyashita, 1991; Wirth et al., 2003), which is a key mechanism for episodic memory formation. In spite of the major significance of these works in advancing our understanding of episodic memory, their contribution has been limited. On the one hand, human studies have not addressed episodic memory formation at the single neuron level. Animal studies, on the other hand, have relied on extensive reward-driven training with numerous repetitions of non-natural stimuli, thus offering a limited account on how single exposures to natural stimuli can give rise to the rapid encoding of new episodic memories.

Neurons in the human MTL have been found to respond to concepts that are related to each other (Quian Quiroga, 2012; Quian Quiroga et al., 2005), such as two co-stars in the same television series or a few researchers (previously unknown to the patients) involved in the experiments (Quian Quiroga et al., 2009; Viskontas et al., 2009). Here we designed a paradigm to study how fast these associations can be created and whether this speed is compatible with basic mechanisms of episodic memory creation. We postulate that associations can be formed by partially overlapping cell assemblies encoding related concepts (Quian Quiroga, 2012) and show experimental evidence of rapid changes of single-cell responses while contextual associations are learned. As detailed below, in order to gain such evidence, we combined the ability to analyze trial-by-trial changes in the robust firing of highly selective MTL neurons (Quian Quiroga et al., 2005, 2008, 2009), with the rapid facility that humans have for learning complex associations and consciously declare them.

**Figure 1:**
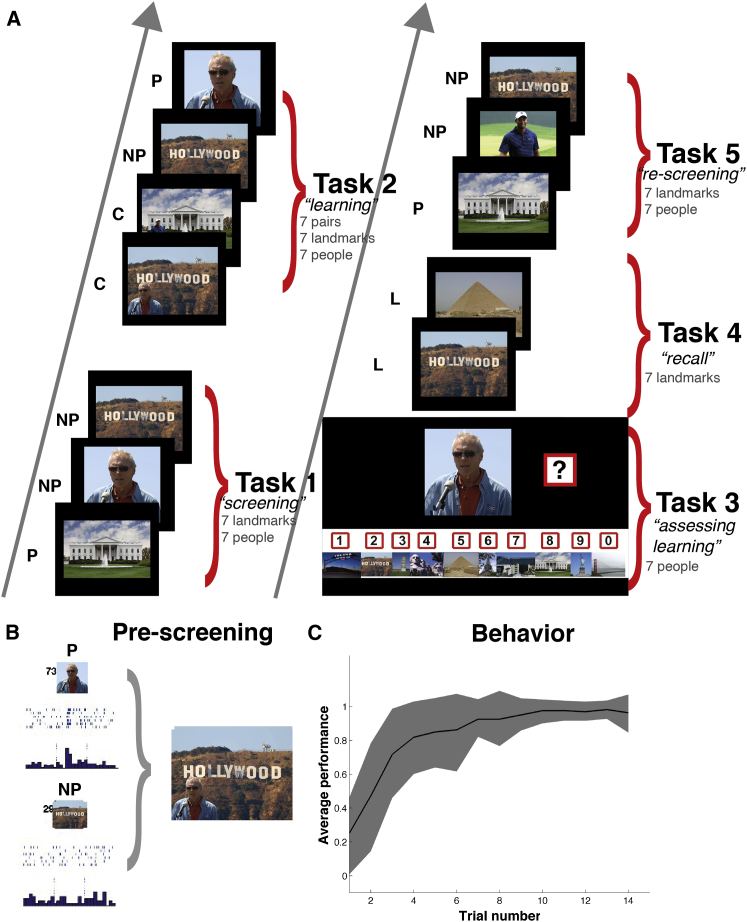
Experimental Design and Behavioral Results (A) Structure of the association task. P, preferred stimulus; NP, non-preferred stimulus; C, composite stimulus; L, landmarks. (B) Selection of stimuli. In this example, in a previous recording session (performed prior to the tasks in A to determine pictures eliciting responses in the neurons), we identified one single unit that responded to a picture of the American actor Clint Eastwood (P) and did not change its firing rate in response to the picture of the Hollywood sign (NP). The preferred (P) and non-preferred (NP) stimuli for each neuron were used to create contextual pictures as the one shown (median 7 pairs, 3–8 pairs per session). (C) Grand average learning curve (mean ± SD) for all pairs in 25 sessions performed by 14 patients. Trial number refers to trials during Task 3, where learning was assessed. Note the high variability across sessions.

Patients first participated in a “screening session” (Quian Quiroga et al., 2005) in which a large number of images of people, animals, and places were presented to find out which (if any) of the recorded neurons responded to a picture. Data processing (spike detection, sorting, and identification of responsive cells) was done quickly (typically within 1 hr) and 3 to 8 (median 7) pairs of pictures were selected. Each pair consisted of a picture of a person (or animal) and a picture of a landmark, for which there was a neuron firing to one of them (the preferred “P” stimulus) and not to the other one (the non-preferred “NP” stimulus). For each pair, we created contextual “composite” images, in which each individual was digitally extracted from the original picture and placed in front of the landmark, mimicking a real photo of seeing the individual at that landmark (Figure 1). Using presentations of the single and composite images of each pair, we evaluated changes in neural activity while subjects performed five consecutive tasks (Figure 1). First, to get an estimation of the pre-learning firing to each picture, in Task 1, the screening was repeated showing each of the single pictures for 1 s 6 times in pseudorandom order, and patients were asked to indicate whether the picture contained a human face or not. Then, a block of “learning and evaluation trials” (median of 15 trials) comprising interleaved tasks 2 and 3 were shown. In Task 2, the composite images (each of them being a specific person in a specific place) were presented in pseudorandom order, which were then followed by the presentation of the single pictures, also in pseudorandom order. The instructions were the same as in Task 1 (i.e., indicate presence of a human face). After each run of Task 2, the learning of associations was tested in Task 3 (the patient was presented each face at a time and had to select the landmark corresponding to it). After Task 2 and Task 3, in Task 4 the patient was presented 6 times each landmark in pseudorandom order and had to name the person that was there. Finally, in Task 5 (“re-screening”) all single pictures were presented again in pseudorandom order, to compare with Task 1 (before learning). Typically, the entire experiment lasted between 25 to 30 min.

## Results

### Firing Patterns of Single Cells during Learning

**Figure 2:**
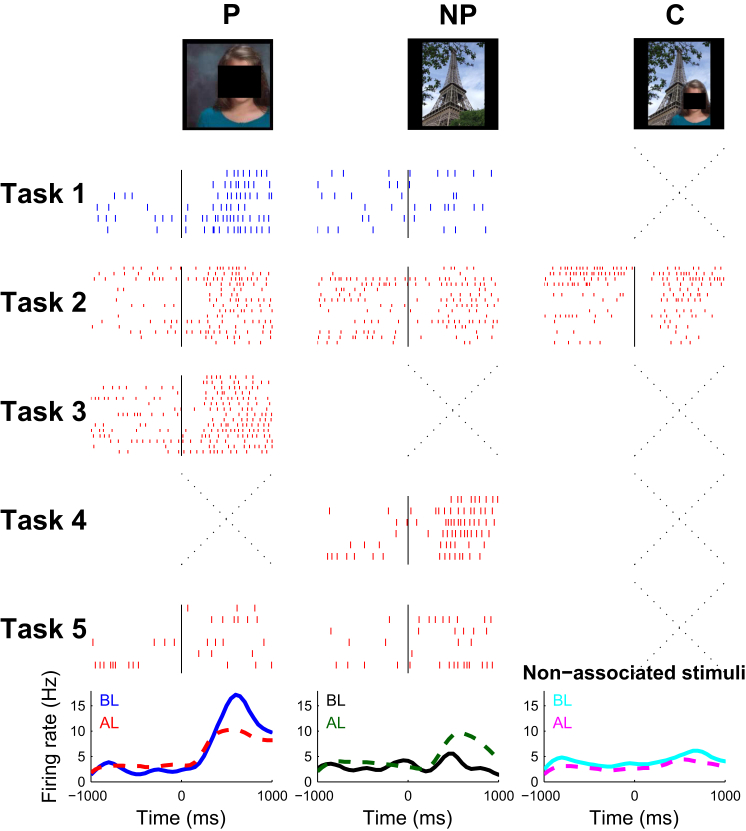
Exemplary Response in the Hippocampus A unit in the left hippocampus of participant 14 was activated with a response of 13.1 spikes/s when the image of the patient’s family was presented (preferred stimulus, black squares have been added for privacy reasons). The same cell was not responsive (response: 3.3 spikes/s) to the image of the Eiffel tower before learning (Task 1). For each task the corresponding raster plots (ordered from top to bottom) of each picture are given. Blue rasters represent pre-learning (Task 1) or incorrect trials. Red rasters represent correct or post-learning (Task 5) trials. The spike density function for trials before (BL) and after (AL) learning in response to the non-preferred (left), preferred (middle), and to the mean of the non-associated stimuli (average over 7 pictures) are shown at the bottom panels. Crosses indicate that the stimulus was not shown during a given task. After single-trial learning (Tasks 2, 3, and 4), the unit fired strongly to the picture of the patient’s family (mean: 10.8 spikes/s, left), to the composite picture (7.8 spikes/s, right) and to the picture of the Eiffel tower (7.6 spikes/s). There was a 230% increase in firing to the non-preferred stimulus. The response to the non-associated stimuli slightly decreased from 5.3 spikes/s before learning to 3.6 spikes/s after learning.

In 14 patients, who participated in 25 experimental sessions (and only 22 for Task 5), we recorded the activity of multiple single neurons using electrodes implanted in the MTL for clinical reasons. Figure 2 shows a neuron in the hippocampus that responded strongly to the picture of a member of the patient’s family (with a mean firing rate of 13.1 spikes/s, SD = 3.9, median = 12.5) but not to the Eiffel tower (3.6 spikes/s on average, SD = 3.4, median = 3.3. The firing to the Eiffel tower during the response period did not differ significantly from the one during baseline (3.9 spikes/s on average, SD = 2.0, median = 4.2), according to a Wilcoxon rank-sum test (p = 0.84, *W* = 40.5, n_1_ = n_2_ = 6). With our experimental design, we aimed to establish whether MTL neurons will widen their tuning to encode the formed association by selectively increasing their firing to the associated stimulus. After a single exposure of the composite picture, the subject learned the association (i.e., family member at the Eiffel tower) and the firing rate in response to the Eiffel tower increased to 7.6 spikes/s on average (SD = 5.1, median = 8.3), a 230% increase compared to the presentations of the Eiffel Tower before learning took place (Task 1). This difference was significant (p = 0.002, *W* = 563, n_1_ = n_2_ = 27, Wilcoxon rank-sum test between baseline and response periods, see Experimental Procedures). In contrast, the response to the preferred stimulus (family member) did not change significantly after learning the association (9.4 spikes/s, SD = 4.5, median = 10.8) and it was similar to the response to the composite image of “family member at the Eiffel tower” (7.8 spikes/s, median = 8.3; p = 0.96, *W* = 325, n_1_ = 27, n_2_ = 15, Wilcoxon rank-sum between the response to the Eiffel tower and the composite image). In order to verify that the increase in firing after learning was specific to the associated stimulus pair (NP) and not common to other stimuli used in the experiment, for example, due to an increase in familiarity, we also examined the response to the other stimuli. For each neuron X with a preferred stimulus Px and a non-preferred stimulus NPx, we defined the non-associated (NA) stimuli for neuron X to be all the other pictures used in the association experiment corresponding to the same category of the NPx stimulus (person or landmark). The bottom-right plot of Figure 2 shows the average response to all the NA stimuli, which decreased from a mean of 5.3 spikes/s (SD = 5.6) to 3.8 spikes/s (SD = 4.9) after learning.

**Figure 3:**
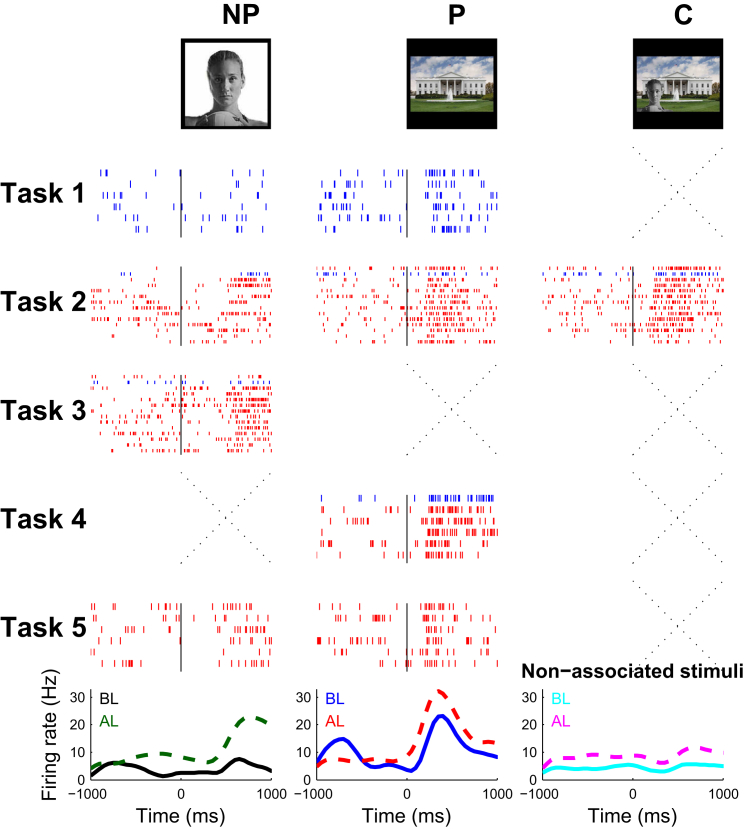
Exemplary Response in the Parahippocampal Cortex Conventions are the same as in Figure 2. A multi-unit in the parahippocampal cortex of participant 3 fired at a rate of 17.8 spikes/s (SD = 7.2) to the picture of the White House (preferred stimulus) from a baseline of 4.4 spikes/s (SD = 4.0). This cell only fired at a rate of 5.0 spikes/s (SD = 3.6) to the picture of the American volleyball player Kerri Walsh before learning (Task 1). After learning (trial 1 in Task 2), the cell selectively increased (by 246%) its response to the pair associate (mean response: 13.8 spikes/s, SD = 9.1, p < 0.05).

For some other units, the association was established the other way around, i.e., a neuron initially responding to a landmark changed its firing to the associated person after learning. Figure 3 shows a multi-unit in the parahippocampal cortex that, in Task 1 (before learning), originally fired to an image of the White House (mean = 17.8 spikes/s, SD = 7.2, median = 15) and not to American beach volleyball player Kerri Walsh (mean = 5.0 spikes/s, SD = 3.6, median = 3.3). After the patient learned the association between these two concepts (trial 1 in Task 2, see Experimental Procedures for learning criterion), there was an increase in the firing of the neuron to the picture of Kerri Walsh (mean = 13.8 spikes/s, SD = 9.2, median = 14.2), which was statistically significant (p < 0.05, Wilcoxon rank-sum test between baseline and response periods). This increase in the neuron’s response to Kerry Walsh (NP stimulus) after learning was observed in all tasks: a mean of 12.9 spikes/s in Task 2 (post-learning trials only), 16.7 spikes/s in Task 3, and 9.4 spikes/s in Task 5. The response to the preferred stimulus (the White House) increased slightly after learning to 25.6 spikes/s (SD = 8.9), but this difference was not significant (Wilcoxon rank-sum test). Additional examples are shown in Figure S1 and Movie S1.

### Population Responses

**Figure 4:**
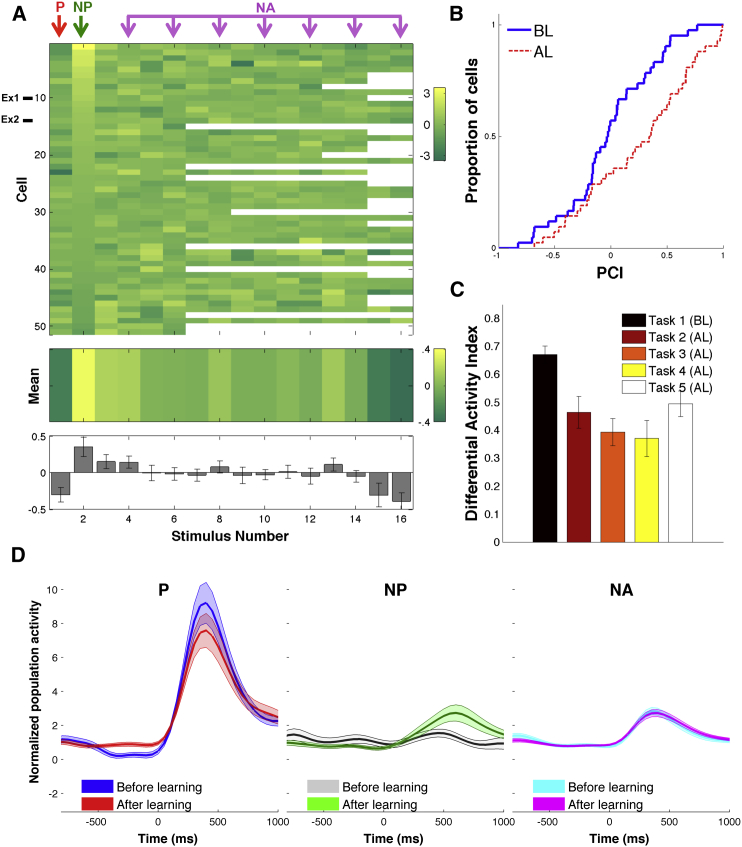
Population: Visually Responsive Units (A) Response changes for all visually responsive units. Each row represents one cell and each column represents one stimulus. The rows were sorted by the strength of the change in the NP stimulus and the columns were unsorted. Blank squares represent stimuli that were not shown during the corresponding session. The mean values across all cells are shown in the middle panel (in colors) and in the bottom panel including SEMs. Ex1, Ex2 correspond to the exemplary units shown in Figures 2 and 3. (B) Cumulative frequency histograms of the correlation coefficient (defined as in Higuchi and Miyashita, 1996) for units before learning (BL) and after learning (AL). Correlation coefficients were significantly higher after learning than before learning (p = 0.007, Kolmogorov-Smirnov test). (C) Average differential activity index DAI = (P_r_ − NP_r_ / P_r_ + NP_r_) for all tasks. Lower values of DAI denote more similar responses. Responses to the preferred and non-preferred stimuli become more similar after learning for all tasks (p < 0.001, average decrease by a factor of 1.6, range: 1.4–1.8). (D) Average normalized spike density function (SDF) for 51 visually responsive units to the P, NP, and NA before and after learning. There was a significant increase in the response strength to the NP stimuli after learning (p < 0.05, Wilcoxon rank-sum test).

We recorded from a total of 613 units (438 multi-units and 175 single units) from the hippocampus (138 units), entorhinal cortex (117 units), amygdala (194 units), and parahippocampal cortex (164 units). We first identified visually responsive units, defined as those that, before learning, showed a significant difference in the response to at least one stimulus using a Wilcoxon rank-sum test between baseline and response (see Experimental Procedures). Altogether, we found 51 visually responsive units (31 single units and 20 multi-units) that significantly increased their firing rate in response to the preferred stimulus (P), with P being one individual (27 units) or landmark (24 units). Figure 4 shows the population results for all visually responsive units. Figure 4A shows the increase in response strength (comparing before and after learning) for each of the 51 visually responsive units and for all stimuli. The population averages are shown at the bottom of Figure 4A for all types of stimuli, where we observe a larger increase in firing after learning for the NP compared to the other stimuli. The change in firing rate after learning (see “Visually Responsive Units” in Experimental Procedures) was significantly different for the different stimuli according to a one-way ANOVA F(11,492) = 3.15, MSE = 0.46, p = 0.0001 (n = 42 cells with at least 12 stimuli—9 units that corresponded to sessions where less than 12 stimuli were presented were excluded from this analysis to avoid unbalanced data). This significant difference was largely due to the change in the NP stimuli and not any other non-associated stimulus. In fact, the difference was still significant when excluding the P stimuli (p = 0.01) but not when also excluding the NP stimuli (p = 0.76). Moreover, the only two stimuli that showed a median significantly different from zero were the preferred stimulus (decrease, p = 0.001; see below for interpretation in terms of repetition suppression) and the NP stimulus (increase, p = 0.005). Furthermore, paired t tests showed that the increases in the NP responses were significantly larger than the ones to any other stimulus (all p values between 0.0008 and 0.03). To further validate these results, we performed a permutation test, adjusted for multiple comparisons, by shuffling the labels of the stimuli and taking as test statistic the smallest difference between the activity to the NP stimulus and the one to any other stimuli. We ran 5,000 permutations and found the p value of the NP stimulus to be statistically significant (p = 0.012, see Supplemental Experimental Procedures for details). Of all the 613 units that we recorded from, 51 were visually responsive and 562 were non-responsive (i.e., did not have a significant response compared to baseline before learning). Of the 562 non-responsive units, 12 (2.1%) exhibited a significant increase to at least one image (mean = 4.1 images, SD = 1.5) after learning took place, according to a Wilcoxon rank-sum test between the baseline and response periods. This number is within what could be expected by chance (n = 28) with a false positive rate of 0.05. Only three of the non-responsive units had a change in response to an association pair (P and NP) that was larger than the one to the other pictures (Wilcoxon rank-sum test, p < 0.05). To further quantify the responses of all visually responsive neurons (to all of the presented stimuli), we calculated a pair-coding index (PCI), a correlation coefficient for each neuron between the mean response to each stimulus and its paired associate (as defined in Higuchi and Miyashita, 1996). This statistic has been used to assess how neurons acquire stimulus selectivity through associative learning and is expected to approach zero for a large number of neurons firing with a pattern independent of the stimulus pairs (Naya et al., 2003). Across the population of visually responsive units, we found that the pair-coding indices after learning (median = 0.35) were significantly higher (median = −0.03, D = 0.36, n_1_ = n_2_ = 42, p = 0.007, Kolmogorov-Smirnov test, see Figure 4B), thus showing the formation of an association between the P and NP stimulus pairs.

To assess the changes that occurred in different tasks, we calculated, for the whole population of visually responsive units, an average differential activity index DAI = (P_r_ − NP_r_ / P_r_ + NP_r_), where P_r_, NP_r_ denote the mean activity in the response interval (see Experimental Procedures). The DAI is expected to be positive, since P_r_ > NP_r_, and it quantifies the difference in the response to the preferred and non-preferred stimuli. As expected, the largest DAI values were obtained for Task 1 (Figure 4C) before learning took place, indicating a large difference in the response to the P and the NP stimuli. For the following tasks, DAI values were significantly smaller (p < 0.001, see Experimental Procedures).

To study the time course of the responses, we separated the normalized population response for all visually responsive neurons according to the type of stimuli (P, NP, and NA) and condition (before and after learning). After learning (Figure 4D), we found a 172% increase in the response strength to the NP stimuli compared to the pre-learning value. This increase was statistically significant (p = 0.05, n = 51, Wilcoxon rank-sum test between the mean response before versus after learning). In contrast, the mean response to the preferred stimuli decreased to 87% of its pre-learning value (p = 0.3, n = 51, Wilcoxon rank-sum test), while the mean response to the non-associated stimuli did almost not change (101% of the pre-learning value, p = 0.9, n = 51, Wilcoxon rank-sum test).

Given these population results, we next evaluated how many of the visually responsive neurons encoded the enforced associations. For this, we defined “pair-coding neurons” as the ones that: (1) showed a significant response to the NP stimulus after learning, using a Wilcoxon rank-sum test comparing baseline and response periods (with p < 0.05), and (2) the distribution of increases of single-trial responses to the NP stimulus after learning was larger than the distribution of increases of single-trial responses to all the other pictures (excluding P) after learning (see “Pair-Coding Units” under Experimental Procedures). Of the 51 visually responsive units, 21 (41%) were “pair-coding neurons” and selectively increased their response to the NP stimuli after learning. As expected by construction—since based on the screening sessions we chose the NP stimuli to be one that the neuron originally did not fire to—these units showed no significant response to the NP stimuli before learning took place (Wilcoxon rank-sum test). The number of neurons encoding the association (pair-coding neurons, n = 21/51) far exceeded the number expected by chance (p < 10^−13^), according to a binomial test with a chance level of 0.05 (see Supplemental Experimental Procedures). We also verified that the observed distribution of p values was significantly lower than the one generated by neurons with a Poisson firing probability and the same mean firing rates as the responsive units (p < 0.004; see “Proportion of Pair-Coding Units” under Supplemental Experimental Procedures).

**Figure 5:**
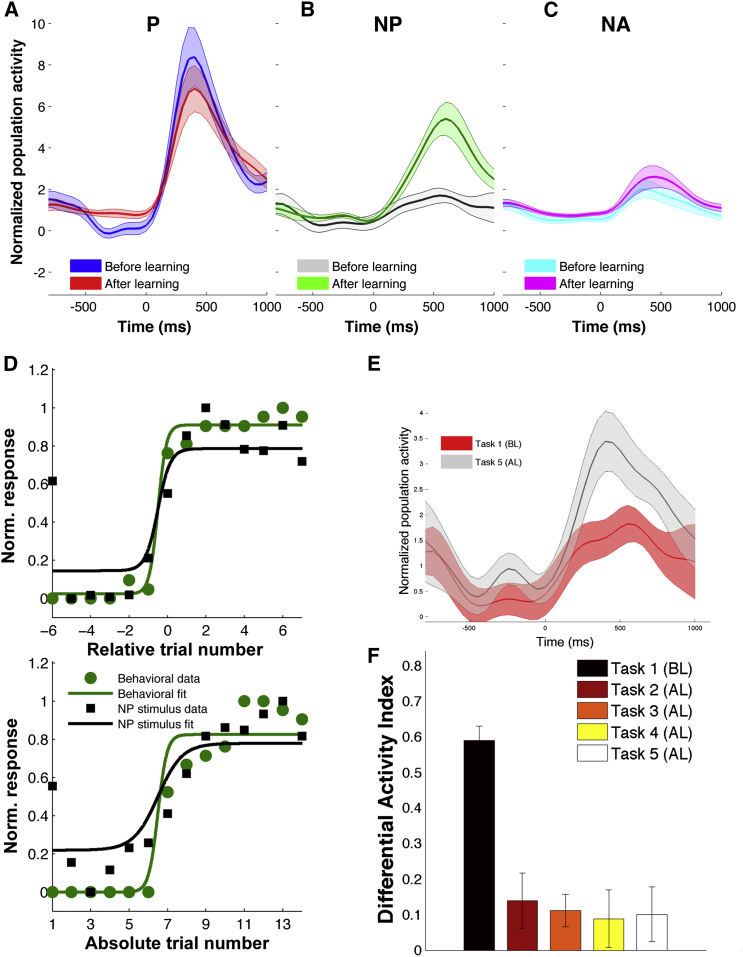
Population: Pair-Coding Units Average normalized spike density function (SDF) for 21 units that selectively changed their response after learning. The shaded areas represent SEM. (A) Normalized SDF to the preferred stimulus for before learning (BL) and after learning (AL). There was no significant difference between conditions in the response period. (B) Normalized SDF to the non-preferred stimulus for BL and AL. After learning, units responded significantly more strongly to the non-preferred stimulus (p < 0.01). (C) Normalized SDF to the non-associated stimuli. (D) Average normalized neural activity (black squares) and behavioral responses (green circles) to the non-preferred stimulus as a function of trial number. In the top panel, data were aligned to the learning time (relative trial number 0). In the bottom panel, trials were sorted according to their presentation order, with the first 6 trials always denoting trial 1 and trial 7 corresponding to the start of Task 2. Continuous lines correspond to psychometric fits using a binomial function. Note that the neural activity follows the sudden increase in behavioral learning when data are aligned relative to learning time. (E) Normalized SDF to the non-preferred stimulus for Task 1 and Task 5. Responses during Task 5 were significantly higher than during Task 1 (p = 0.001, Wilcoxon rank-sum test). (F) Average differential activity index DAI for all tasks. Responses to the preferred and non-preferred stimuli become more similar after learning for all tasks (p < 10^−6^, average decrease by a factor of 5.5, range: 4.2–6.7).

In what follows, we concentrate on the 21 neurons that encoded the associations. Among the 21 pair-coding units, 14 (67%) originally fired to a person and started firing to the associated landmark after learning (like the one shown in Figure 2). In the remaining 7/21 cases, the association was established the other way around, i.e., the neuron originally responding to a landmark, changed its firing to the associated individual after learning (like the one shown in Figure 3). Across the population of pair-coding neurons, the responses to the non-preferred stimuli showed an average increase of 281% (from 1.44 ± 0.22 to 4.06 ± 0 0.38, mean ± SEM) after learning, which was statistically significant (p < 10^−5^; Wilcoxon rank-sum test between the mean response before versus after learning) (Figure 5B). Similar results were obtained when considering only single units (n = 11). In this case, there was a significant increase of 412% in the response to the NP stimuli after learning (p = 0.0001, Wilcoxon rank-sum test). In line with the results for all visually responsive units (Figure 4C), the responses to the P (Figure 5A) and NA (Figure 5C) stimuli did not change significantly after learning (88% of the pre-learning value, p = 0.65 for the P stimulus and 134% of the pre-learning value, p = 0.27 for the NA stimuli).

### Neuronal and Behavioral Learning Curves

In order to compare on a trial-by-trial basis the neural and behavioral changes, we calculated the neuronal learning curves by rescaling the activity across the population of neurons encoding the association for all trials in all tasks to the range 0–1 (see Experimental Procedures). A direct comparison between the behavioral and neural learning curves exhibited a significant positive correlation for the non-preferred stimulus (r = 0.25, p < 10^−11^, Pearson’s correlation coefficient r), due to the increase in firing after learning the associations. There was a non-significant correlation for the non-associated stimuli (r = 0.05, p = 0.2) and also a negative correlation for the preferred stimulus (r = −0.08, p = 0.03), consistent with the decrease in firing to the preferred stimulus reported in Figure 4A, which is likely due to repetition suppression in line to a previous work without an association paradigm (Pedreira et al., 2010). To further investigate whether this behavior is due to repetition suppression, or whether it also reflects the formation of associations, we compared the decreases found in pair-coding units with the ones found in the other visually responsive units. For this, for each visually responsive unit, we calculated the percentage change as 100^∗^(P_post_ − P_pre_)/P_pre_, where P_post_ and P_pre_ indicate the mean activity in the response window. Both populations of pair-coding and non-pair-coding units exhibited similar trends, with a mean percentage decrease of −7% (SD = 40, median = −15%) for the pair-coding units and −11% (SD = 33, median = −20%) for the non-pair-coding units. The median of both populations did not differ significantly (p = 0.64, Wilcoxon rank-sum test) and the median of both populations differed significantly from zero (p = 0.03 and p = 0.02 for pair-coding and non-pair-coding, respectively), thus the decreases for the P stimuli seem to reflect repetition suppression rather than an encoding of the association.

Given the variability on the number of trials that subjects needed to learn each association pair, to further evaluate how tightly correlated were the observed firing changes to the actual learning of the associations, we realigned the response of each cell to the learning time and compared these neural responses to the ones obtained as a function of the actual trial number in the experimental session. For this, we fitted logistic functions to the average behavioral and neural learning curves to the non-preferred stimulus for both alignments (absolute trials and relative to learning time; see Experimental Procedures for details). In Figure 5D, we first observe that aligning to learning time gives a more accurate matching between the behavioral and the neural learning curves. In fact, for data aligned to learning there were no significant differences between behavioral and scaled neural data (Kolmogorov-Smirnov test, p = 0.59). Additionally, the fits were more accurate for data aligned to learning time, as quantified by the Akaike Information Criterion (AIC) (Akaike, 1974)—data relative to learning: AIC^Beh^ = 142; AIC^Neu^ = 298; data not aligned: AIC^Beh^ = 175; AIC^Neu^ = 325, where smaller values denote higher accuracy. The same logistic model did not fit the data for the preferred and non-associated stimuli as accurately as it did with the non-preferred stimuli (Figure S2). Moreover, both for the P and NA, Pearson’s correlation coefficient was larger for the unaligned (R^2^ = 0.73/0.04 for P/NA stimuli) than for the learning-aligned data (R^2^ = 0.46/0.03). When the NP data were aligned to learning time, there was a large increase in the slope of the behavior curve: β^Beh^ = 4.6 after re-alignment, compared to β^Beh^ = 4.2 for the unaligned data. But the interesting fact was that this change in the behavioral learning curve was accompanied by an abrupt increase in firing to the non-preferred stimuli, when re-aligning the neural data to the learning time: β^Neu^ = 3.1 aligned to learning, compared to β^Neu^ = 1.6 without alignment. The slope difference (with and without alignment to learning) was significant, according to a non-parametric bootstrap test (p < 0.05; see Figure S3 and Experimental Procedures for details).

### Neural Activity during Different Tasks

Next, to rule out that changes in neuronal responses were just driven by one of the specific tasks we used (as each task varied in complexity and attentional demand) and not by the formation of new associations, we used the differential activity index DAI introduced before for the population of visually responsive units. Altogether, after learning there was a decrease of DAI values by a factor of 5.5 on average (range: 4.2–6.7). Moreover, differences between the tasks *after learning* were not significant, thus suggesting that these neuronal changes were not task dependent. Supporting this view, a direct comparison of the response to the non-preferred stimuli in the two identical tasks (Task 1, pre-screening and Task 5, re-screening) showed significant differences (p = 0.001, Figure 5E), which can be attributed to the learning of the particular association. There was also an increase in the response during Task 5 when considering all visually responsive units but in this case the difference was not significant (p = 0.14).

### Decoding Analysis

From a readout viewpoint, the learning of the associations should be accompanied by a decrease in the discriminability between the NP and the P stimulus, given that the neuron originally firing only to the P stimulus starts also firing to the NP after learning. This selective increase in firing to the NP stimuli should also lead to more discriminability between the NP and NA stimuli after learning. This is indeed what we observed using a linear classifier to decode the identity of the stimuli before and after learning (see Supplemental Experimental Procedures). When considering the whole population of visually responsive units, the discrimination between P and NP stimuli went down from a 74% average performance before learning to 68% after learning. The decrease was significant according to a paired t test, t(100) = 1.95, p = 0.03. For pair-coding units, the discrimination between P and NP stimuli went down from a 72% average performance before learning to 56% after learning. Altogether, the decoding performance was significantly larger than chance with p < 0.05 (see Supplemental Experimental Procedures) for 11 of the 21 responses (52%) before learning and for 6 of the 21 responses after learning (38%).

### Latency Analysis

Two possible mechanisms can in principle account for the increased response to the NP stimuli after learning. On the one hand, neurons can rapidly change their tuning and start firing to the NP stimuli directly—that means, a neuron originally encoding the P stimulus starts encoding the NP stimulus after learning—in which case, the time courses of both P and NP signals are expected to be similar. On the other hand, the NP stimuli can act as a cue to evoke the representation of (and in turn the neuron’s firing to) the P stimuli. Following previous works (Naya et al., 2001, 2003), we distinguished between these two putative mechanisms—namely between Type 1 and Type 2 neurons—by analyzing the differences in the latency response onsets between the NP and P stimuli. In the first case (Type 1), we expect similar latency onsets for the P and NP stimuli, whereas in the other case (Type 2), we expect a larger latency onset for the NP stimuli. We used Poisson spike train analysis (see Experimental Procedures) to estimate the onset latency for all presentations and performed a Wilcoxon rank-sum test to compare the latency values for the P and NP stimuli. Of the 21 pair-coding units that selectively increased their firing to the NP stimuli after learning, 13 were “Type 1,” as in the example shown in Figure 2, and the remaining 8 were “Type 2,” as in the example shown in Figure 3. The scatter plot of the response onset latency values with the classification details is shown in Figure S4. Interestingly, both Type 1 and Type 2 units exhibited a significant positive correlation between behavioral performance and neural activity for the NP stimulus (Pearson’s r = 0.24, p = 10^−7^ and r = 0.28, p = 4^∗^10^−6^ for Type 1 and Type 2, respectively).

### Regional Analysis

Altogether, we identified 51 visually responsive units across different regions within the MTL: 10 in hippocampus, 7 in the entorhinal cortex, 29 in the parahippocampal cortex, and 5 in the amygdala. We observed pair-coding units throughout the MTL: (6 out of 10 [60%] visually responsive units in the hippocampus), 4 out of 7 (57%) in the entorhinal cortex, 11 out of 29 (38%) in the parahippocampal cortex, and 1 out of 5 (40%) in the amygdala. We consistently found both Type 1 and Type 2 neurons in these regions: 4 out of 8 pair-coding units in H/EC were of Type 1, where we have grouped responses in hippocampus and entorhinal cortex that were previously shown to exhibit similar properties (Mormann et al., 2008; Quian Quiroga et al., 2009). In PHC, 7 out of 11 pair-coding units were of Type 1. Pair-coding cells in H/EC were more prominently firing to pictures of persons instead of landmarks (6 out of 8 pair-coding units) compared to cells in PHC (n = 7 out of 11) but the difference was not significant (χ^2^ = 0.28, p = 0.60). Despite the small sample size of the recorded neurons, we found that the time courses of the responses in PHC were qualitatively similar to the ones in H/EC (Figure S5). As a cautionary note, we wish to point out that a larger number of recorded neurons is necessary to address the issue of regional differences (and similarities) more conclusively.

## Discussion

Episodic memory—the ability to consciously recall personal experienced events and situations (Moscovitch, 1994; Tulving, 2002)—relies on the very rapid and effortless formation of new associations (Bunsey and Eichenbaum, 1996; Quian Quiroga, 2012; Wirth et al., 2003; Kahana et al., 2008). Animal studies have previously shown that single neurons can change their selectivity after learning in associative tasks (Erickson and Desimone, 1999; Gochin et al., 1994; Messinger et al., 2001; Sakai and Miyashita, 1991; Wirth et al., 2003). In particular, Miyashita and colleagues trained macaque monkeys to associate pairs of fractal patterns and found picture-selective neurons in IT cortex (areas TE and perirhinal cortex) that showed significantly correlated responses to the paired associates (Sakai and Miyashita, 1991). This coding was later hypothesized to emerge from separate TE neurons coding perceptual information about the individual paired associates that would converge onto the same neurons in the perirhinal cortex (the selective-convergence model) (Higuchi and Miyashita, 1996; Naya et al., 2001, 2003). But the learning of paired associates in animals is a demanding task that requires extensive reward-driven training, typically taking place before recordings begin (Erickson and Desimone, 1999; Higuchi and Miyashita, 1996; Sakai and Miyashita, 1991). Moreover, these recordings were performed in extra-hippocampal regions, which show distributed representations and are not thought to support fast learning according to modeling studies (McClelland et al., 1995). One notable exception was reported by Wirth and colleagues (Wirth et al., 2003; Yanike et al., 2004), who demonstrated a significant correlation between behavioral performance and neuronal hippocampal activity during the acquisition of associations between background scenes and specific actions (a saccade toward one of four cardinal locations). However, in this case the task also involved explicit reward-driven training, and learning occurred in two-thirds of the cases only after 14–17 trials (Wirth et al., 2009). These timescales are longer than the ones concomitant with episodic memory, which is seemingly effortless and often triggered by single presentations.

Besides the need of reward-driven training, a major caveat to develop animal models of episodic memory is the lack of verbal or complex feedback to assess conscious recollection. In an earlier study, we showed that neurons in the human MTL respond in a reliable and specific manner during viewing of video episodes such as a clip of *The Simpsons* and also during the free conscious recall of that same clip (Gelbard-Sagiv et al., 2008). Human MTL neurons have also been reported to act as novelty and familiarity detectors (Rutishauser et al., 2006). A recent work (Miller et al., 2013) has studied modulations in the firing of place-responsive neurons in the human MTL while subjects learned item-location associations during a virtual navigation task followed by free recall. The authors calculated a neural similarity index between the ensemble activity of these place cells during navigation and during item recall and found that such index was higher for the ensemble of place cells near the location of the item. Considering the previous finding that MTL neurons show an invariant representation of concepts (Quian Quiroga et al., 2005), our results of association formation in these neurons suggest conceptual associations. In particular, we show: (1) the encoding of associations at the single-cell level, (2) the learning of the associations on a trial-by-trial basis (showing the emergence of robust responses at the exact moment of learning), (3) the precise latency of the responses, distinguishing two type of neurons, (4) the neurons’ responses in different tasks, including free recall, also comparing the exact same task before and after learning (Task 1 versus Task 5), (5) that these changes were specific to the associated (compared to the other non-associated) stimuli, and (6) a decoding approach provided differences in discrimination performance after learning consistent with our other analyses. Overall, by showing that such associations can be created with arbitrary but conceptually coherent concepts (i.e., persons in particular scenes, in contrast to pair association tasks in which two arbitrary pictures are associated), our results provide strong evidence pointing toward a role of the MTL beyond a spatial representation of the environment. Moreover, the emergence of associations of concepts established after single trials linked to rapid neural activity changes is ideal for the creation of new episodic memories (Quian Quiroga, 2012).

How different MTL regions contribute to episodic memory formation is still a subject of intense discussion (Diana et al., 2007; Eichenbaum et al., 2007). Neuroimaging works have advocated that episodic encoding is mediated by the hippocampus, which supports the relational binding of the individual elements to the context of an episode (see Davachi, 2006; Quamme et al., 2007), and the parahippocampal cortex, which is involved in item memory (Kirwan and Stark, 2004) and/or in relational memory (Diana et al., 2007). The PHC has been shown to be involved in both spatial (Buffalo et al., 2006) and nonspatial contextual associations (Aminoff et al., 2007; Law et al., 2005). Related lesion studies in animals have suggested that the hippocampus is important for item-item associations, while parahippocampal cortex is critical for recognition memory for object-place associations (Higuchi and Miyashita, 1996; Malkova and Mishkin, 2003). In line with these studies, we found pair-coding units not only in H/EC (8/21) but also in PHC (11/21).

A long-lasting debate in the psychology literature (Roediger and Arnold, 2012), refers to whether the formation of associations occurs gradually (Hull, 1943) or all-or-none (Estes et al., 1960; Rock, 1957). In the first case, the strength of association between each pair develops gradually until the first item produces a recall of the second. This assumes that learning reflects a continuous buildup of the strength of memory traces. Alternatively, association pairs could be learned at once and repeated trials are just giving several opportunities for the formation of the association (Estes et al., 1960). In line with the latter view, the formation of associations to NP stimuli changed abruptly, with a large increase in the slope of both the behavioral and neural learning curves, thus supporting all-or-none learning. A majority of neurons that changed their tuning after learning had a similar response-onset latency for the P and NP stimuli (Type 1), thus suggesting the creation of associations by combining distinct concepts through partially overlapping representations—namely, some of the neurons initially encoding one concept started firing to the associated one after learning (Quian Quiroga, 2012). One could in principle relate gradual learning with Type 2 neurons—in other words, the later response onset latency of NP compared to P may imply a recall of P when showing NP. However, learning occurred in a median of 1 trial for Type 2 neurons (and a median of 2 trials for Type 1 neurons). So, rather than supporting gradual learning, Type 2 neurons could be showing an evoked representation based on associations created using partial overlapping representations by Type 1 neurons (and it is also plausible to expect that after more repetitions the latency difference between P and NP may disappear). Importantly, associations were encoded by widening the tuning of neurons previously encoding one of the concepts, rather than by recruiting new neurons encoding each association because less than 1% of the initially non-responsive units started firing to a pair of associated images, compared to a 41% of visually responsive neurons that expanded their tuning to encode the associations.

Due to clinical constrains and day-to-day variability of the recordings, it is currently not possible to assess how the changes in neuronal tuning reported here may evolve at longer timescales (days, weeks, months, or years) to establish long-term memories and a robust encoding of related concepts. It seems, however, reasonable to postulate that this initial encoding of associations, established after single presentations, may be further consolidated with time in some cases but may also disappear in others, considering that a relatively large proportion of neurons encoded the associations. Although the inception of episodic memories—like remembering the context and sequence of salient events when meeting a friend at a particular café—goes beyond the formation of contextual associations, our study suggests a fundamental mechanism of neuronal plasticity that may support episodic memory formation.

## Experimental Procedures

### Subjects

14 patients with pharmacologically intractable epilepsy (10 right handed, 6 male, 18 to 53 years old) participated in this study. Patients were implanted with chronic depth electrodes for 7–10 days to determine the seizure focus for possible surgical resection. The number and specific sites of electrode implantation were determined exclusively on clinical grounds and were verified by MRI or by computer tomography co-registered to preoperative MRI. Patients volunteered for the study and gave written informed consent. The study conformed to the guidelines of the Medical Institutional Review Board at UCLA.

### Electrophysiology

Each electrode contained nine platinum-iridium microwires at their end. Eight of the microwires acted as the active recording electrodes and the ninth microwire acted as a reference. The differential signal from the microwires was amplified and filtered between 1 and 9,000 Hz. Data from six patients were recorded with a 64-channel Neuralynx system with a sampling rate of 28 kHz. In the remaining eight patients, data were acquired at 30 kHz using a 128-channel acquisition system (Blackrock Microsystems). The extracellular signals were band-pass filtered (300 Hz to 3 kHz) and later analyzed offline. Spikes were detected and sorted using wave_clus (Quian Quiroga et al., 2004). Single- and multi-unit activity was classified by one of the authors (M.J.I.) based on spike shape, variance, and the presence of a refractory period for the single units (i.e., <1% spikes within <3 ms interspike interval distributions) (Quian Quiroga et al., 2005).

### Experimental Sessions

Subjects sat in bed facing a laptop computer on which pictures were presented. In the screening sessions, they were instructed to respond whether the image showed a person or not with a button press. Approximately 105 pictures were displayed six times in pseudorandom order (Ison et al., 2011; Quian Quiroga et al., 2005).

In each recording session, a median of 8 (range: 2–28) of the recorded neurons responded to one or more pictures. Each of these responsive neurons fired to a median of 2 pictures (range 1–18 stimuli), which gives an average selectivity of 2.6% (range: 0.8%–30%), in agreement with values reported in a previous study showing an invariant representation by these neurons (Quian Quiroga et al., 2005). After each screening session, we selected a subset of the stimuli (mean: 14, range: 6–16) to create the images to be shown in the “association sessions,” as depicted in Figure 1 and described in the main text. Further information about these sessions can be also found in the Supplemental Experimental Procedures. All the methods described below correspond to the analyses of the association sessions.

### Analysis of the Neural Data

#### Visually Responsive Units

For each image presentation, we considered two intervals based on the response latency of neurons recorded from the medial temporal lobe (Mormann et al., 2008): a baseline interval starting 500 ms before stimulus onset and ending 100 ms after stimulus onset and a response interval between 200 ms and 800 ms after stimulus onset. Responses were defined as the median firing rate in a segment (baseline/response) across trials (Quian Quiroga et al., 2005). We identified visually responsive units as those that significantly responded to at least one individual or landmark before learning took place. The criterion for significance of the response was based on a Wilcoxon rank-sum test (with p < 0.05) between the baseline and response periods and we additionally required a median firing rate of at least 2 Hz following stimulus onset. For each stimulus presented (P, NP, and others), we quantified the firing rate changes after learning for stimulus “i” as Delta_i = Resp_i(AL) − Resp_i(BL), using a *Z* score normalization for each unit and phase (BL/AL): Resp_i = (mean(FR_i) − mean(FR))/SD(FR).

#### Pair-Coding Units

We defined pair-coding units as the ones that selectively changed their response to the associated picture after learning (see below for the definition of learning time), fulfilling the following criteria: (1) they had a significant increase in the response to the NP stimulus (the paired associate of the preferred stimulus) with respect to baseline after learning (Wilcoxon rank-sum test), and a non-significant response to NP before learning (Wilcoxon rank-sum test), and (2) the distribution of single trial increases after learning (i.e., subtracting the mean number of spikes before learning in the response window) for the NP stimulus was significantly larger than the distribution of single trial increases after learning for all the other pictures (excluding P) according to a Wilcoxon rank-sum test across trials.

#### Pair-Coding Index

We also used a pair-coding index defined using a correlation coefficient as in Higuchi and Miyashita (1996): $CC=\sum \left[\left(x_{i}-\mu \right)\left(x_{i^{\prime }}-\mu^{\prime }\right)\right]/\sum {\left\{\left[{\left(x_{i}-\mu \right)}^{2}\right]\left[{\left(x_{i^{\prime }}-\mu^{\prime }\right)}^{2}\right]\right\}}^{1/2}\left(i=1-12\right)$, where x_i_ denotes the mean response for the i-th stimulus, and the i’-th pictures are the ones belonging to the associated pair, μ and μ’ are the averages of x_i_ and x_i’_. This calculation was done over n = 42 visually responsive units that correspond to sessions where at least 12 stimuli were shown.

#### Comparisons between Conditions

In the examples shown in Figures 2 and 3 and Figure S1, we used the raw data (number of spikes in the response window) and Wilcoxon rank-sum tests to compare between different conditions. For comparing the population responses before and after learning, we used normalized data (see “Time Courses of Behavioral and Neural Data”) and Wilcoxon rank-sum tests between responses before and after learning.

#### Time Courses of Behavioral and Neural Data

To study the time course of the responses, we built the spike density function by convolving each spike train with a Gaussian kernel (width = 100 ms). For the analyses at the population level we normalized the firing rates for each neuron by calculating a *Z* score for each 50 ms width bin: $z=FR_{response}-FR_{baseline}/SD_{baseline}+\eta$, where *FR*_*response*_ is the smoothed firing rate in the bin, *FR*_*baseline*_ is the mean firing rate during the baseline period, *SD*_*baseline*_ is the standard deviation of firing rates averaged for all trials, and η = 0.1 is a regularization term. We obtained the normalized population response by averaging the *Z* scores of a given neuron in response to a stimulus type (preferred, non-preferred, non-associated) and averaging over all the trials depending on the analysis (e.g., pre-learning trials, post-learning trials, all trials in a given task).

#### Differential Activity Index

To quantify the difference in firing in the different tasks, we computed a differential activity index DAI = (P_r_ − NP_r_ / P_r_ + NP_r_), considering the mean activity in the response interval of the normalized response (where P_r_ and NP_r_ are the mean normalized responses to the preferred [P] and nonpreferred [NP] stimuli, respectively). We used z tests to assess the significance of the difference in the DAI across different tasks (Figure 5F).

#### Latency Estimation

Onset latencies for responsive units were determined by Poisson spike train analysis (Hanes et al., 1995; Mormann et al., 2008). To compare the latency values for the P and NP stimuli, we estimated the onset latency for all presentations and then performed a Wilcoxon rank-sum test. This procedure allowed us to separate the neurons into Type 1 neurons, which fired to the P and NP stimuli with a latency that was not significantly different (Wilcoxon rank-sum test and interquartile range < 250 ms), and Type 2 neurons, which showed a significantly longer latency to the NP compared to the P stimulus.

#### Behavioral Learning Curves

We calculated the learning curves for individual picture pairs and subjects. For each paired associate, we annotated whether each response was correct or incorrect for all the trials of Task 3 (in which subjects had to identify the landmark where each person was). Subjects performed a median of 15 trials (range: 14–19), where each trial corresponds to a complete cycle through the entire set of stimuli used in the task. We estimated the trial where learning occurred by fitting the behavioral learning curves with a logistic function:

(Equation 1) $$f\left(x\right)=\frac{1-\gamma -\lambda }{1+\exp\left(-\beta \left(x-\alpha \right)\right)}+\gamma$$

where α corresponds to the threshold, *β* denotes the slope of the logistic function (low values of beta correspond to gradual transitions and high values of beta correspond to abrupt transitions), and *λ,γ* are two parameters related to the pre-learning lower asymptote (*γ*) and post-learning upper asymptote (1 – λ). We used a Maximum Likelihood Criterion to estimate the optimal parameters and obtained the learning time from the closest trial following α (the threshold *f*(*x* = α) = 0.5, for *λ = γ = 0*). All subjects learned most pairs (mean: 98.3%) but the learning time varied across subjects. The learning criterion was reached on average after 2.9 trials (median 2, interquartile interval: 2).

#### Comparison of Neural and Behavioral Learning Curves

To allow a comparison with the behavioral fits, the neural data were smoothed and rescaled to a range of 0–1. For this, we rescaled the neural activity (N) to the range 0–1 (N_r_) using $N_{r}=N-\min\left(N\right)/\max\left(N\right)-\min\left(N\right)$. We then measured the similarity between neural and behavioral learning curves with a Pearson’s correlation coefficient. To further quantify whether the changes in the neural activity were gradual or sudden, we fitted the neural learning curves with logistic functions with β as the only free parameter (Equation 1). The values of λ,γ were taken from the pre-learning and post-learning firing rates/behavioral performance, where 0/1 corresponds to pre/post-learning, respectively. The threshold α, calculated for each individual pair, was kept constant. For the data aligned to absolute trial number, we considered the first 14 trials in chronological order (which corresponded to presentations during Tasks 1, 2, and 3).

#### Assessing the Quality of the Fits

We evaluated the quality of the fits following an information theoretic approach by means of the Akaike Information Criterion (Akaike, 1974). The lower the value of AIC, the more accurate the fit. To test the significance of the difference in the parameters (slope, AIC) for the neural data with different alignments, we performed a non-parametric bootstrap procedure (Kingdom and Prins, 2010).

## Author Contributions

M.J.I., R.Q.Q., and I.F. designed the electrophysiology study; I.F. performed the surgeries; M.J.I. collected the electrophysiological data; M.J.I. analyzed the data; M.J.I., R.Q.Q., and I.F. wrote the paper. R.Q.Q. and I.F. contributed equally to the study. All authors discussed the results and implications and commented on the manuscript at all stages.

## Supplemental Information

Document S1. Supplemental Experimental Procedures, Figures S1–S5, and Table S1

Movie S1, Related to Figure 3. An Example of a Single Neuron Firing during Learning of New Associations The movie has three parts. The first part shows a unit that fires preferentially to a picture of the White House before the associated pairs are presented. Due to time constraints, responses to only some of the stimuli are presented. Part two shows the response of the same neuron when associations are created. The picture of the White House (preferred stimulus) is associated to the picture of the beach volleyball player Kerri Walsh (non-preferred stimulus). The video shows how the cell becomes responsive to the picture of Kerri Walsh after learning. Part three shows a summary of the results for the exemplary unit and also for the population of cells. MPEG animation (.mp4) 6.5 MB.

Document S2. Article plus Supplemental Information
