# Supplementary material for: Rapid Encoding of New Memories by Individual Neurons in the Human Brain
Source: Neuron. 2015 Jul 1;87(1):220–30. doi: 10.1016/j.neuron.2015.06.016 (PMC4509714; doi:10.1016/j.neuron.2015.06.016)
Supplement: Document S1. Supplemental Experimental Procedures, Figures S1–S5, and Table S1 [file mmc1.pdf]

Neuron

Supplemental Information

Rapid Encoding of New Memories

by Individual Neurons in the Human Brain

Matias J. Ison, Rodrigo Quian Quiroga, and Itzhak Fried

Supplemental Figures and Legends

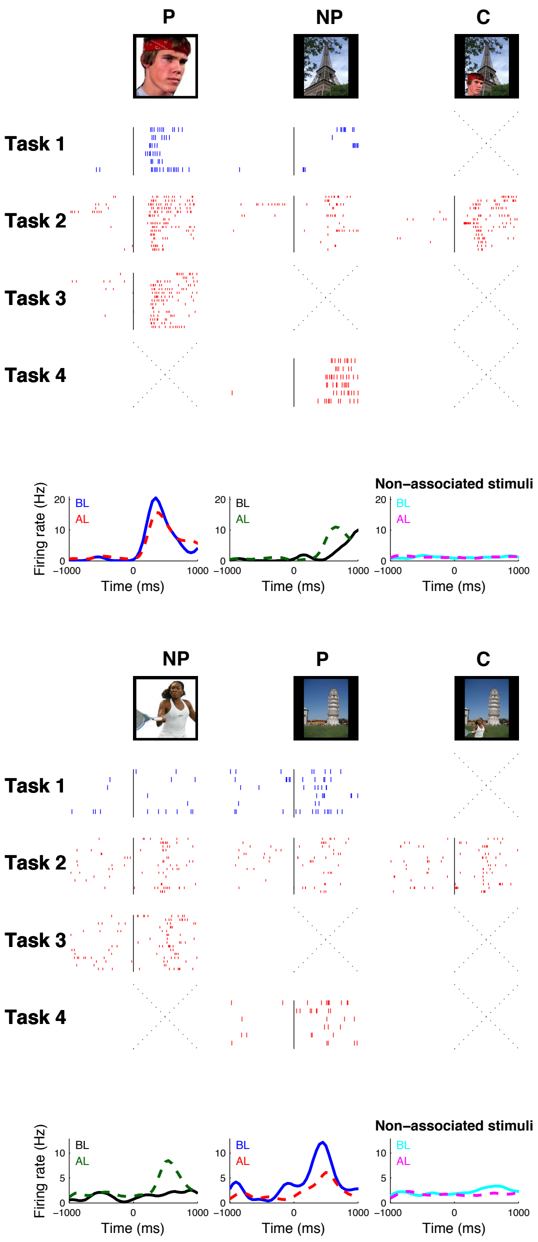

Figure S1, Related to Figure 2. Example of single unit responses: Conventions are the same as in Figure 2. **Top panel)** A unit in the right hippocampus initialy fired to an image of the actor Josh Brolin before learning (mean=14.2 spikes/s, s.d.=6.3, median=11.7) and did not fire to the image of the Eiffel tower (mean=1.7 spikes/s, s.d.=3.3, median=0). After learning the unit fired strongly to the picture of Josh Brolin (mean: 11.4 spikes/s, left panels), to the composite picture (9.8 spikes/s, right panels) and to the picture of the Eiffel tower (6.3 spikes/s). There was a 370% increase in firing to the non-preferred stimulus. The cell did not fired to the non-preferred non-associated stimuli before learning (1.1 spikes/s, s.d.=2.8) nor it did so after learning (1.1 spikes/s, s.d.=2.4). **Bottom panel**). A cell in the left parahippocampal cortex that initially responded to the picture of the Pisa tower (mean = 8.06, s.d. = 3.9) and not to the picture of the tennis player Venus Williams (mean = 1.9. s.d. = 2.5) . After learning this unit fired significantly to both images (Venus Williams: mean = 5.5, s.d. = 3.4; Pisa tower: mean = 4.3, s.d. = 3.1).

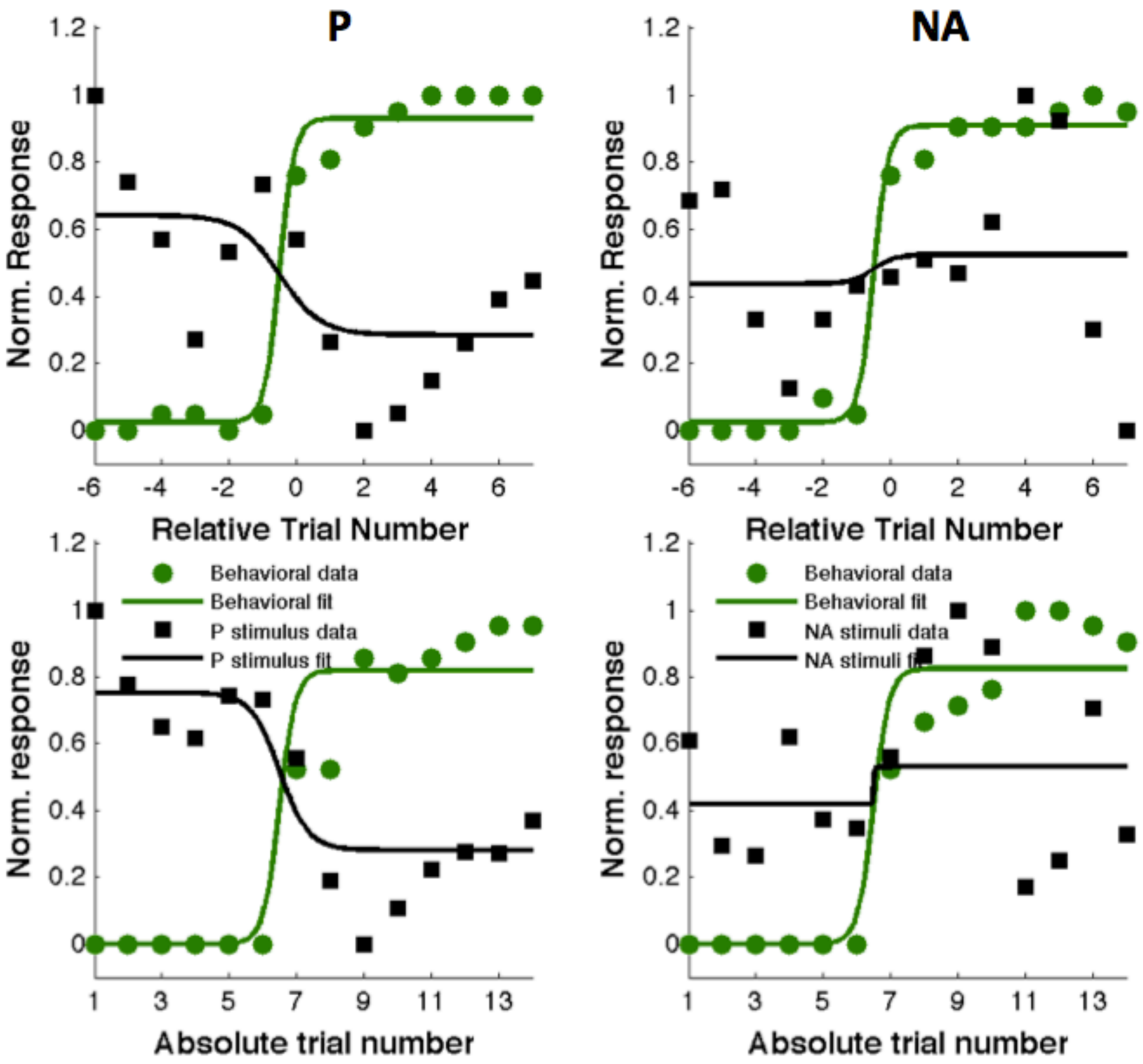

Figure S2, Related to Figure 5D. Neural and behavioral responses to the preferred (P) and non-associated (NA) stimuli. Average normalized neural activity (black squares) and behavioral responses (green circles) to the P stimulus (left panel) and NA stimuli (right panel) as a function of trial number. Conventions as in Figure 4D. For P, since the average response to the preferred stimuli decreased throughout the experiment, the fits were done on the reversed data (which corresponds to negative values of β). Please note that neural data were rescaled to the range 0-1 to allow comparison with the behavioral responses. The figures show that the logistic model did not fit the data accurately. Pearson's coefficient R^2=0.46 for P stimuli (data aligned to learning), R^2=0.73 (for unaligned data); NA stimuli: R^2=0.03 data aligned to learning, R^2=0.04 unaligned data.

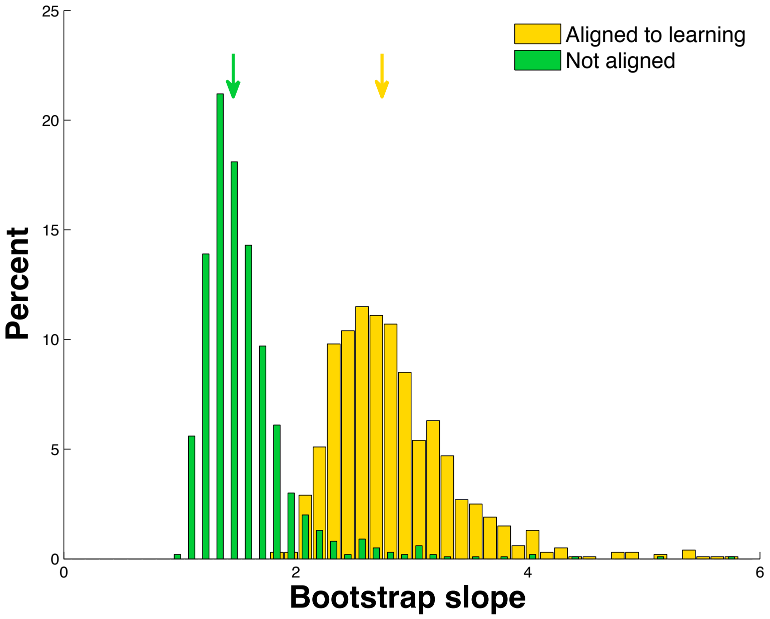

Figure S3, Related to Figure 5D. **Bootstrap analysis of the learning curves.** To test the significance of the difference in the slopes for the neural data's learning curves with different alignments (see Figure 5D), we performed a non-parametric bootstrap procedure. We generated 1000 samples based on the observed data for each condition (data aligned to learning, not aligned). For each simulation we fit the data and obtained bootstrap slopes. The difference in the slope was obtained for each run and we estimated the p value of the difference by determining the fraction of times that the observed difference was less than 0, which was observed in 22/1000 simulations, thus yielding a p value of 0.02. The medians of the distributions are indicated with arrows.

.

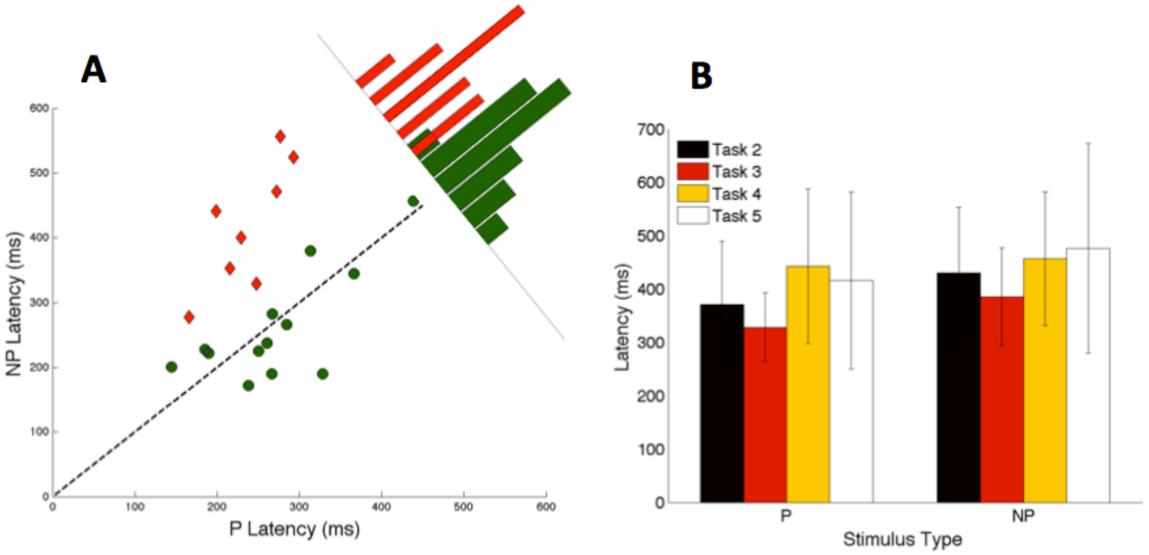

Figure S4, Related to Figure 5A. Latency analysis. (A) Scatter plot of the response onset latency values for the preferred and non-preferred stimuli for each neuron. Neurons were classified into Type 1 (green) and type 2 (red) according to the onset difference between the P and NP stimuli (see text for details). Type 1 neurons (n=13) fired to the P and NP stimuli with similar latency (P>0.05 Wilcoxon rank-sum test and interquartile range <250 ms). Type 2 neurons (n=8) had a significantly longer latency for the NP compared to the P stimulus (P<0.05 Wilcoxon rank-sum test). The Pearson's correlation coefficient for type 1 neurons was 0.75. We projected this scatter plot onto the diagonal (equal latencies). The resulting distribution was bimodal with a median significantly different from zero (P=0.04, Wilcoxon signed-rank test). (B) Average latency for the P and NP stimuli in all tasks after learning. The latency values across the population of cells obtained from all the post learning tasks did not differ (ANOVA, F(3,53)=0.69/1.67, P= 0.56/0.18 for NP/P, respectively). Error bars denote SD.

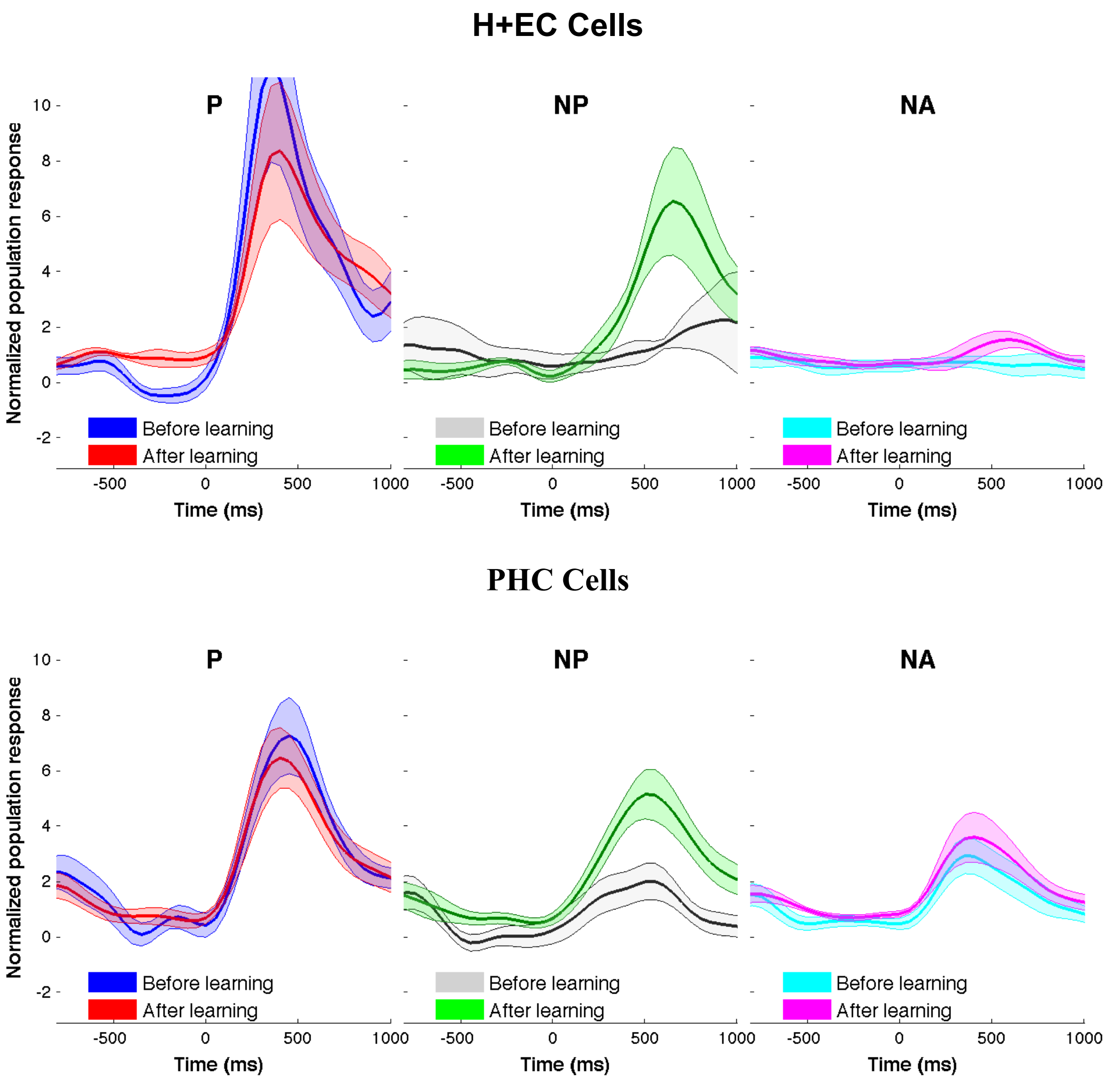

Figure S5, Related to Figure 5A. Population response for different MTL areas. Average normalized spike density function for pair-coding units in the Hippocampus and Entorhinal cortex (N=8) (top panel) and for pair-coding units recorded from the parahippocampal cortex (N=11). Cells recorded from both regions showed similar behavior (no significant changes in the preferred stimulus and large significant increase in the response to the non-preferred associated stimulus).

Table S1: List of Experimental Details per patient

Patient ID	Sex	Age	# sessions	# pairs	# trials in Task 2	# vis resp units	# pair-coding units
1	F	37	2	(7,8)	(15,15)	8	6
2	M	37	2	(7,7)	(15,15)	9	2
3	F	22	2	(8,8)	(14,14)	6	3
4	F	45	2	(8,8)	(14,14)	2	1
5	M	34	2	(3,3)	(14,14)	3	0
6	M	40	1	7	14	1	1
7	F	52	1	7	16	0	0
8	F	19	3	(8,8,8)	(15,15,19)	9	4
9	F	53	2	(7,5)	(15,15)	2	0
10	M	27	1	8	15	2	1
11	F	18	1	4	15	0	0
12	M	45	2	(6,6)	(15,15)	2	1
13	M	29	2	(3,3)	(15,15)	6	1
14	F	50	2	(7,7)	(15,15)	1	1
N=14	8F	37*	2*	7*	15*	N=51	N=21

# pairs: (x,y): Number of pairs used in each session.

***denotes median value over row**

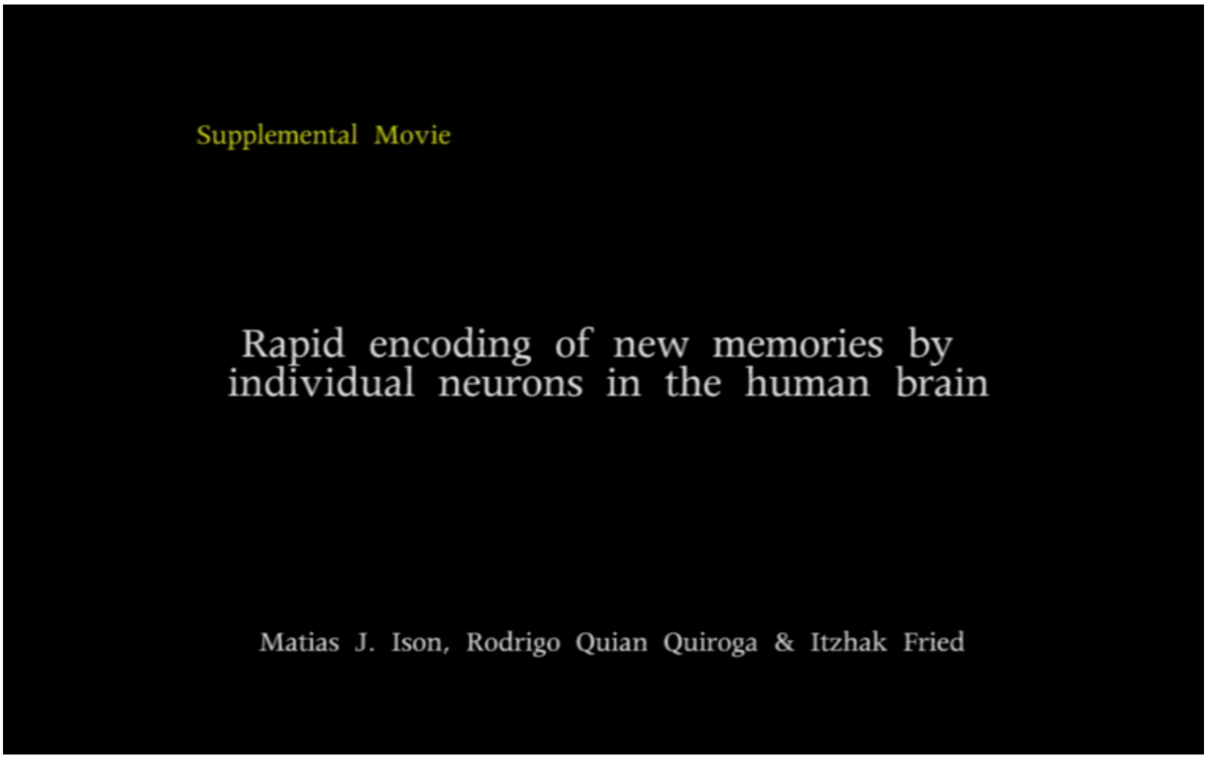

Movie S1, Related to Figure 3. An example of a single neuron firing during learning of new associations. The movie has three parts. The first part shows a unit that fires preferentially to a picture of the White House before the associated pairs are presented. Due to time constraints, responses to only some of the stimuli are presented. Part two shows the response of the same neuron when associations are created. The picture of the White House (preferred stimulus) is associated to the picture of the beach volleyball player Kerri Walsh (non-preferred stimulus). The video shows how the cell becomes responsive to the picture of Kerri Walsh after learning. Part three shows a summary of the results for the exemplary unit and also for the population of cells.

MPEG animation (.mp4) 6.5 MB

Supplemental Experimental Procedures

Subjects. 14 patients with pharmacologically intractable epilepsy (10 right handed, 6 male, 18 to 53 years old) participated in this study. Before starting the sessions, subjects were explained the different experimental tasks, including that they would see pictures of people appearing at different places and they would be asked to recall these associations later. The study conformed to the guidelines of the Medical Institutional Review Board at UCLA.

Electrophysiology. Electrodes contained nine platinum-iridium microwires (40 μm) protruding ~4 mm into the tissue beyond the tip of the electrode. Eight of the microwires acted as the active recording electrodes (impedance = ~200-500 kΩ) and the ninth microwire was blunted and acted as a low-impedance (~2-5 Ω) reference. The differential signal from the microwires was amplified and filtered between 1 and 9000Hz. Data from 6 patients were recorded with a 64-channel Neuralynx system (Bozeman, MT) with a sampling rate of 28 kHz. In the remaining 8 patients, data were acquired at 30 kHz using a 128-channel acquisition system (Blackrock Microsystems, Salt Lake City, UT). The extracellular signals were bandpass filtered (300 Hz to 3 kHz) and later analyzed offline. Spikes were detected and sorted using wave_clus (Quian Quiroga et al., 2004).

Experimental sessions. All paradigms were implemented in MATLAB (Mathworks, Natick, MA) using the psychophysics toolbox (Brainard, 1997). Subjects sat in bed facing a laptop computer on which pictures were presented. In the screening session, they were instructed to respond whether the image showed a person or not with a button press. A median of 105 pictures (interquartile range: 96-114) were displayed six times in pseudorandom order (Ison et al., 2011; Quian Quiroga et al., 2005). Each image was shown for 1 second. After each screening session, we selected a subset of the stimuli (mean: 14, range: 6-16) to create the images to be shown in the "association sessions". The number of association pairs were decided ad hoc based on the overall performance of the subjects in the screening sessions and in previous association sessions (were more than one association session was performed in the same subject). The rationale for deciding the number of association pairs in each case was to tune the level of difficulty of the task (trying to make it challenging but not way too difficult for the subject) and the total time required to do the experiment based on the response times of the subject (trying not to go over about 30'). Each image was shown for 1 second and the mean interstimulus interval was 1,910ms (SD=320). Data come from 25 experimental sessions in 14 patients. 3 experimental sessions were completed for tasks 1-4 and 22 for tasks 1-5. Data from 4 additional patients (6 sessions) were excluded due to sessions being interrupted (N=1), lack of well isolated units (N=4) or lack of behavioral learning (N=1).

Data analysis: Neural data. All analyses were performed using custom programs written in MATLAB (Mathworks, Natick, MA).

Proportion of pair-coding units: To test whether the number of observed pair-coding neurons exceeded the one expected by chance we used a binomial test (Fried et al., 2011; Wang et al., 2014). Let N_s indicate the total number of visually responsive units and N_r indicate the number of units that showed a significant change in the response after learning (pair-coding units). Considering the probability of observing the effect in one cell at a chance level of 0.05, the probability of observing N_r or more pair-coding units under the null hypothesis can be calculated with a binomial test. As an alternative scenario, we compared the p-values obtained experimentally against those expected assuming the null hypothesis of a Poisson process (Fried et al., 2011). For each unit we created a simulated spike train with the same firing rate but with the spike times governed by a homogeneous Poisson process. We generated 1,000 simulations, used the same methods and criteria applied to the real data, and found that the simulated p-values were lower than the ones observed in real data in less than 0.4% of the cases.

Proportion of persons preferred units: To test for equality of proportions of units firing preferentially to pictures of persons rather than landmarks in different brain regions we used a chi-squared test, taken together units recorded in the hippocampus-entorhinal cortex against units recorded from the parahippocampal cortex.

Changes in NP stimulus against other stimuli: To assess statistical significance of the increase in the NP responses with learning against the ones to any other stimuli across the population of visually responsive units we used a permutation test. We took the smallest difference in the increase in the mean NP response against all the rest of the stimuli and shuffled the labels of the stimuli. We ran 5,000 permutations and determined the p-value as the fraction of permutations which showed a smallest difference larger than the original test statistic. We observed that this happened 61/5000 times, which gives P=0.012.

Decoding analysis: We used a linear classifier (Fisher's linear discriminant) to decode the identity of the stimuli before and after learning. We took the number of spikes in the response interval as a classifying feature and used a leave-one-out cross-validation, testing for all the pair-coding cells in the before learning and after learning trials. To assess the statistical significance of the decoding performance (relative to the chance level of 0.5) we used permutation tests by randomly rearranging all the labels of the observed data points to obtain the null hypothesis. We used 1,000 permutations and rejected the null hypothesis if the performance of the permuted data was larger than the one observed for more than 5% of the cases.

Latency estimation: Onset latencies for responsive units were determined by Poisson spike train analysis (Hanes et al., 1995; Mormann et al., 2008). For each stimulus, we determined the time between stimulus onset and the onset of the first spike train in all presentations. The median across all presentations was taken as response latency. As in previous studies (Mormann et al., 2008), in cases where the baseline firing rate was <2 Hz or the Poisson method failed to find consistent responses (responses for which less than half the trials closest to the median were >200 ms apart), the median of the first spike during the interval [100 ms 800 ms] was used instead. To compare the latency values for the P and NP stimuli we estimated the onset latency for all presentations and then performed a Wilcoxon rank-sum test. This procedure allowed us to separate the neurons into Type 1 neurons, which fired to the P and NP stimuli with a similar latency (P>0.05 Wilcoxon rank-sum test and interquartile range <250 ms), and Type 2 neurons, that showed a significantly longer latency to the NP compared to the P stimulus.

Assessing the quality of the fits: We evaluated the quality of the fits following an information theoretic approach by means of the Akaike Information Criterion(Akaike, 1974). The AIC can be obtained from the likelihood of the data (L) as $AIC = -2\ln(L) + 2k$, where k represents the number of free parameters in the model. The lower the value of AIC the more accurate the fit. The analysis of the psychometric function was performed using the Palamedes toolbox for MATLAB (Kingdom and Prins, 2010). To test the significance of the difference in the parameters (slope, AIC) for the neural data with different alignments we performed a non-parametric bootstrap procedure (Kingdom and Prins, 2010). We generated 1000 samples based on the observed data for each condition (data aligned to learning, not aligned). For each simulation we fit the data and obtained bootstrap slopes. The difference in the slope was obtained for each run and we estimated the p value of the difference by determining the fraction of times that the observed difference was less than 0.

Stability of multi-units: To quantify the stability of the spike multi-units waveforms we separated the spikes fired during the first five minutes of the recording from the ones that were fired during the last five minutes. To evaluate the similarity of different spike shapes we calculated Pearson's correlation coefficient between the two averages of the waveforms, so that a value of 1 would indicate identical spike shapes (Jackson and Fetz, 2007). Similarities were calculated between pair-coding multi-units during the first/last five minutes of the recording and also between all pairs of different multi-units. The similarity between the same multi-units showed a median of 0.9992) and differed significantly ($P<10^{-4}$, Wilcoxon rank-sum test) from the similarity between different multi-units, which showed a median of 0.9474. For the ISIs, we calculated the mean ISI during the first and last five minutes of the recording for each multi-unit. Although differences in the ISI distribution could arise as a result of changes in unit composition and also as a result of the change in the firing pattern after learning, we found no significant differences between the ISIs during the first and last five minutes (P=0.89, Wilcoxon rank-sum test). Altogether, this shows that

the units that we recorded from did not change their properties over the timescale of the experiment.

Supplemental References

Akaike, H. (1974). A new look at the statistical model identification. Automatic Control, IEEE Transactions on 19, 716-723.
Brainard, D.H. (1997). The psychophysics toolbox. Spatial Vision 10, 433-436.
Fried, I., Mukamel, R., and Kreiman, G. (2011). Internally generated preactivation of single neurons in human medial frontal cortex predicts volition. Neuron 69, 548-562.
Hanes, D.P., Thompson, K.G., and Schall, J.D. (1995). Relationship of presaccadic activity in frontal eye field and supplementary eye field to saccade initiation in macaque: Poisson spike train analysis. Exp Brain Res 103, 85-96.
Ison, M.J., Mormann, F., Cerf, M., Koch, C., Fried, I., and Quiroga, R.Q. (2011). Selectivity of pyramidal cells and interneurons in the human medial temporal lobe. J Neurophysiol 106, 1713-1721.
Jackson, A., and Fetz, E.E. (2007). Compact movable microwire array for long-term chronic unit recording in cerebral cortex of primates. Journal of Neurophysiology 98, 3109-3118.
Kingdom, F.A.A., and Prins, N. (2010). Psychophysics: A Practical Introduction (London: Academic Press, Elsevier).
Mormann, F., Kornblith, S., Quiroga, R.Q., Kraskov, A., Cerf, M., Fried, I., and Koch, C. (2008). Latency and selectivity of single neurons indicate hierarchical processing in the human medial temporal lobe. J Neurosci 28, 8865-8872.
Quian Quiroga, R., Nadasdy, Z., and Ben-Shaul, Y. (2004). Unsupervised spike detection and sorting with wavelets and superparamagnetic clustering. Neural Comput 16, 1661-1687.
Quian Quiroga, R., Reddy, L., Kreiman, G., Koch, C., and Fried, I. (2005). Invariant visual representation by single neurons in the human brain. Nature 435, 1102-1107.
Wang, S., Tudusciuc, O., Mamelak, A.N., Ross, I.B., Adolphs, R., and Rutishauser, U. (2014). Neurons in the human amygdala selective for perceived emotion. Proc Natl Acad Sci U S A 111, E3110-3119.
