# Supplementary figures and images for: Rapid Encoding of New Memories by Individual Neurons in the Human Brain
Source: Neuron. 2015 Jul 1;87(1):220–30. doi: 10.1016/j.neuron.2015.06.016 (PMC4509714; doi:10.1016/j.neuron.2015.06.016)

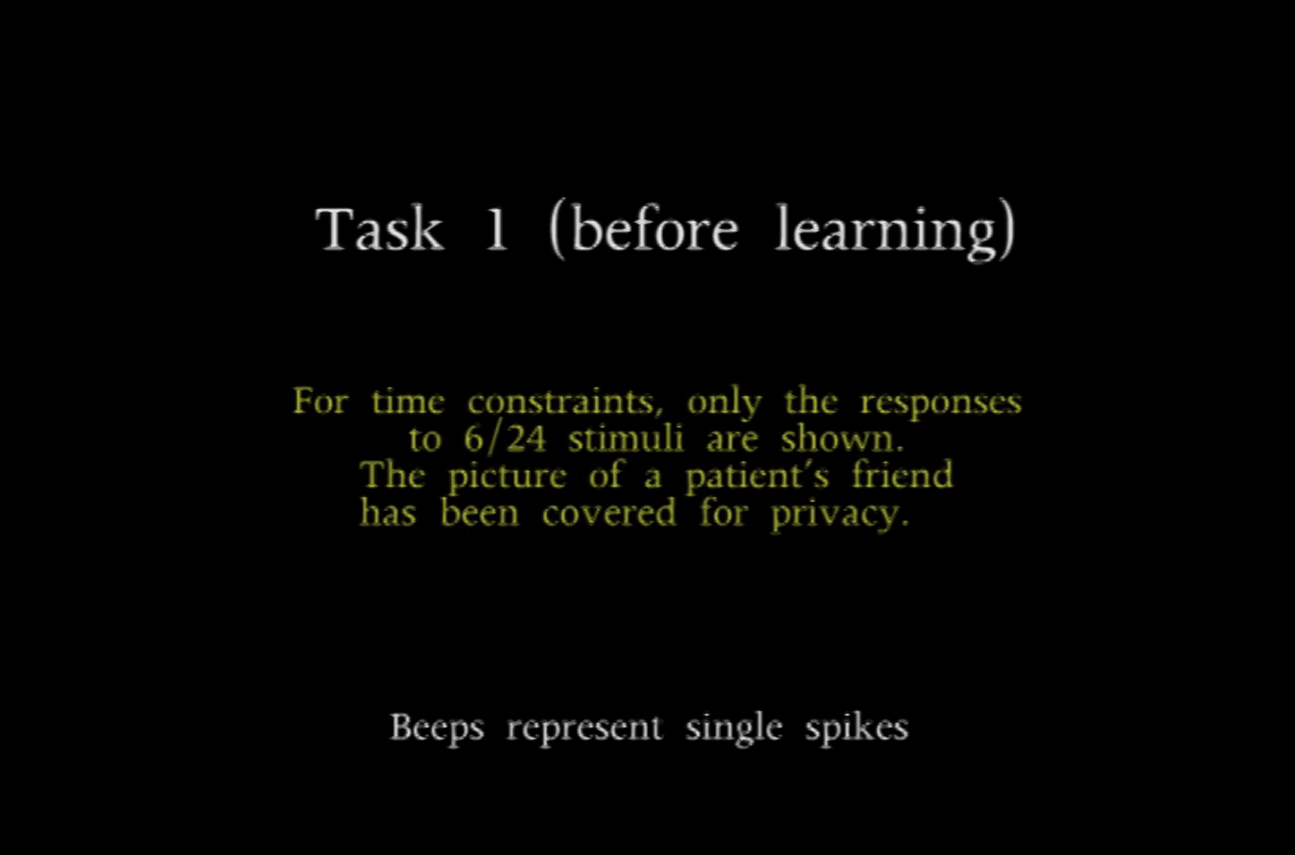

Supplement: Movie S1, Related to Figure 3. An Example of a Single Neuron Firing during Learning of New Associations — The movie has three parts. The first part shows a unit that fires preferentially to a picture of the White House before the associated pairs are presented. Due to time constraints, responses to only some of the stimuli are presented. Part two shows the response of the same neuron when associations are created. The picture of the White House (preferred stimulus) is associated to the picture of the beach volleyball player Kerri Walsh (non-preferred stimulus). The video shows how the cell becomes responsive to the picture of Kerri Walsh after learning. Part three shows a summary of the results for the exemplary unit and also for the population of cells. MPEG animation (.mp4) 6.5 MB. [file mmc2.jpg]
